# Pulmonary Vessel Obstruction Does Not Correlate with Severity of Pulmonary Embolism

**DOI:** 10.3390/jcm8050584

**Published:** 2019-04-28

**Authors:** Marianne Lerche, Nikolaos Bailis, Mideia Akritidou, Hans Jonas Meyer, Alexey Surov

**Affiliations:** 1 Department of Respiratory Medicine, University of Leipzig, Liebigstr. 20, 04103 Leipzig, Germany; Marianne.Lerche@medizin.uni-leipzig.de; 2 Department of Radiology, University of Leipzig, Liebigstr. 20, 04103 Leipzig, Germany; Nikolaos.Bailis@medizin.uni-leipzig.de (N.B.); medula80@gmail.com (M.A.); hans-jonas.meyer@medizin.uni-leipzig.de (H.J.M.)

**Keywords:** pulmonary embolism, Mastora score, CT

## Abstract

The aim of the present study was to analyze possible relationships between pulmonary vessel obstruction and clinically relevant parameters and scores in patients with pulmonary embolism (PE). Overall, 246 patients (48.8% women and 51.2% men) with a mean age of 64.0 ± 17.1 years were involved in the retrospective study. The following clinical scores were calculated in the patients: Wells score, Geneva score, and pulmonary embolism severity index (PESI) score. Levels of D-dimer (µg/mL), lactate, pH, troponin, and N-terminal natriuretic peptide (BNP, pg/mL) were acquired. Thrombotic obstruction of the pulmonary arteries was quantified according to Mastora score. The data collected were evaluated by means of descriptive statistics. Spearman’s correlation coefficient was used to analyze associations between the investigated parameters. *P* values < 0.05 were taken to indicate statistical significance. Mastora score correlated weakly with lactate level and tended to correlate with D-dimer and BNP levels. No other clinical or serological parameters correlated significantly with clot burden. Thrombotic obstruction of pulmonary vessels did not correlate with clinical severity of PE.

## 1. Introduction

Acute pulmonary embolism (PE) is a common disease with a high mortality. According to the literature, the short-term mortality after an episode of acute PE can reach up to 65% [1,2]. Therefore, an immediate risk stratification of patients with acute PE at the time of presentation is very important. The use of pulmonary vessel obstruction quantified by several scores as a possible clinical predictor in PE has been widely discussed in the literature. However, the role of obstruction grade in PE for the prediction of possible complications is still unknown. The reported data regarding associations between pulmonary vessel obstruction and morbidity/mortality in PE are very contradictory. For example, Wu et al. [3] found that clot burden quantified on CT pulmonary angiography was an important predictor of death in patients with PE. Similar results were also reported by Van der Meer et al. [4]. However, other authors did not find any associations between clot burden and mortality in PE [5,6]. 

According to the literature, there are several clinical and serological parameters which have been established as predictors for morbidity and mortality in PE. These include pulmonary embolism severity index (PESI) [7,8], and different serological parameters, such as lactate [9], troponin [10,11] and N-terminal natriuretic peptide (BNP) [12]. Presumably, pulmonary vessel obstruction may correlate strongly with severity of PE. Therefore, the aim of the present study was to analyze possible relationships between pulmonary vessel obstruction and clinically relevant parameters and scores in patients with PE.

## 2. Materials and Methods

This retrospective study was approved by the institutional review board (Nr.: 118/19-ck, Ethics Committee, University of Leipzig, Leipzig, Germany).

Overall, 246 cases with PE were acquired for this retrospective study. There were 120 (48.8%) female patients and 126 (51.2%) male patients with a mean age of 64.0 ± 17.1 years, a median age of 65 years, and a range of 15–97 years. 

The following clinical scores were calculated in the patients: Wells score, Geneva score, and PESI score. Furthermore, D-dimer level (µg/mL), lactate (venous blood, mmol/L), pH (venous blood), troponin (pg/mL), and N-terminal natriuretic peptide (BNP, pg/mL) were acquired for the study.

Additionally, a risk stratification of PE was performed according to the American Heart Association (AHA) as follows: low risk PE, submassive PE and massive PE [13]. 

**Figure 1:**
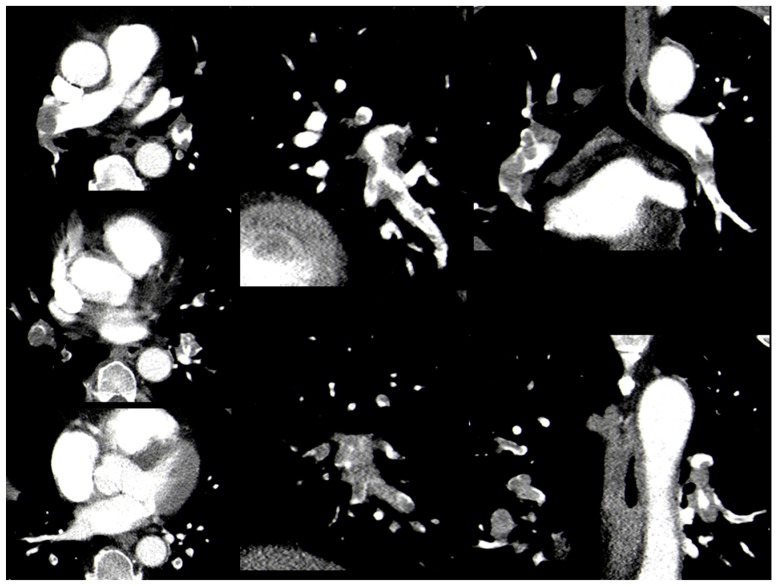
Case with bilateral pulmonary embolism. Total Mastora score is 65%.

Thrombotic obstruction of the pulmonary arteries was calculated according to Mastora et al. [14] (Mastora score), as reported previously. For this score, the obstruction of the mediastinal, lobar, and segmental arteries was quantified by a percentage, i.e., (thrombus divided by the vessel lumen) multiplied by 100%. The analysis of thrombotic obstruction was performed in three-dimensional images (Figure 1). Thereafter, the sum of the percentages of all arteries was calculated as the global obstruction score with a maximum of 300%. 

The statistical analysis and graphics creation were performed using GraphPad Prism (GraphPad Software, La Jolla, CA, USA). Collected data were evaluated by means of descriptive statistics (absolute and relative frequencies). Spearman’s correlation coefficient (*p*) was used to analyze associations between investigated parameters. In all instances, *p* values <0.05 were taken to indicate statistical significance.

## 3. Results

**Figure 2:**
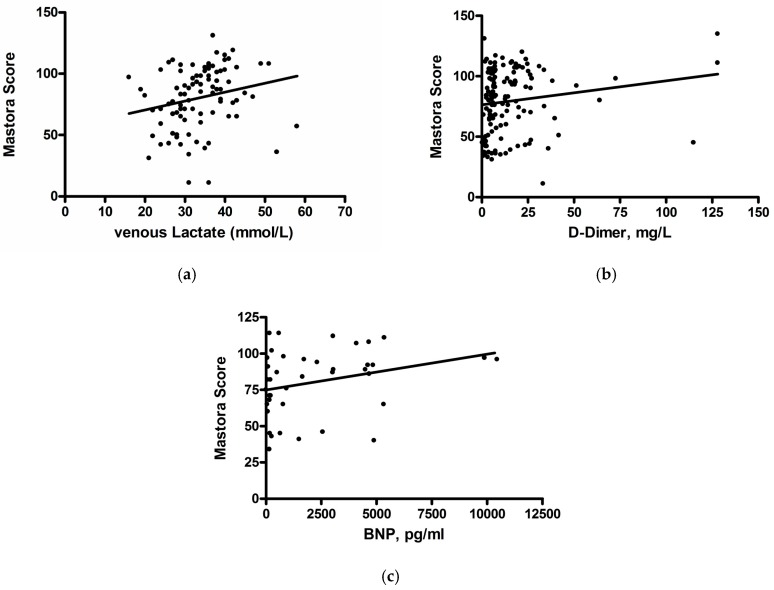
Associations between Mastora score and lactate level (**a**), D-dimer (**b**), and BNP (**c**).

A complete overview of the results including mean values, standard deviation and ranges is shown in Table 1. The results of correlation analysis are given in Table 2. Mastora score correlated weakly with lactate level (ρ = 0.17, *p* = 0.01) and tended to correlate weakly with D-dimer level and BNP (ρ = 0.15, *p* = 0.09 and ρ = 0.29, *p* = 0.063, respectively) (Figure 2a–c). No other clinical or serological parameters correlated statistically significantly with clot burden. 

**Figure 3:**
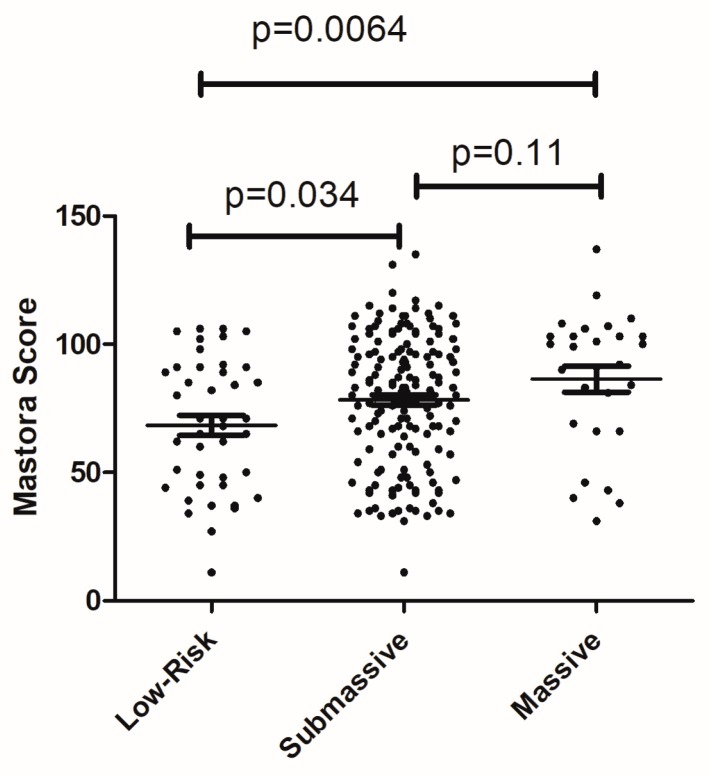
Comparison of Mastora score values between patients with low risk pulmonary embolism (PE), submassive and massive PE.

Furthermore, clot burden between the subgroups of PE according to the AHA was compared. Low risk PE was found in 42 patients, submassive PE in 160 cases, and massive PE in 29 patients. Mastora score values overlapped between the three groups (Figure 3). Patients with massive PE had a higher clot burden in comparison to the patients with low risk PE (*p* = 0.0064). Additionally, in patients with submassive PE, Mastora score values were also higher than in patients with low risk PE (*p* = 0.034). 

## 4. Discussion

This is the first study to analyze direct associations between quantified thrombotic vessel obstruction and clinically relevant parameters in PE. Our results showed that clot burden did not correlate with important clinical and/or serological parameters in PE. This finding is very unusual. In fact, presumably, the severity of pulmonary vessel obstruction may be associated with the severity of clinical manifestation of PE. Interestingly, vessel obstruction also did not correlate with scores predicting probability of PE like Wells and Geneva scores. Previous studies analyzed only the values of the scores in relation to presence/absence of PE but not their associations with the severity of pulmonary vessel obstruction. Furthermore, our study showed that D-dimer level only tended to correlate weakly with Mastora score. Although this finding is also surprising, similar results were reported by other authors [15]. 

Our study also showed that thrombotic vessel obstruction did not correlate with clinically established scores for the prediction of PE severity, such as PESI score. This finding is very important. According to the literature, PESI can accurately stratify risks in PE [16,17]. Furthermore, PESI can predict mortality rate in patients with PE [16]. Our results suggest that thrombotic vessel obstruction may not significantly influence severity and mortality in PE. This is in agreement with some previous reports, which also did not find any associations between clot burden and outcome in PE. To date, Ghuysen et al. [18] have shown that neither the pulmonary obstruction index nor the pulmonary artery pressure in PE could predict patient outcome. According to Apfaltrer et al. [19], clot burden did not correlate with clinical outcome in PE. Similar results were also observed by Furlan et al. [20]. Overall, the data indicated that thrombus burden cannot be used as a clinical marker in PE. However, Attina et al. [21] found that vessel obstruction can be used for a risk stratification of pulmonary heart disease or death in patients with acute PE. Also, in the study of Martinez et al. [22] survivors had lower thrombotic burden in comparison to the deceased patients. 

We found that Mastora score values were different in patients with low risk, submassive and massive PEs according to the AHA risk stratification. However, the values of clot burden overlapped significantly between the subgroups. Therefore, clot burden cannot be used as a certain factor for risk stratification in PE. 

Another important question is the possible relationship between thrombotic vessel obstruction and right ventricular dysfunction (RVD) in PE. Previously, numerous reports found significant associations between RVD and mortality in PE [4,18,19,23]. PE results in a rapid increase in pulmonary vascular resistance that may lead to RVD and eventually to heart failure and death [19]. Presumably, thrombotic vessel obstruction provokes pulmonary arterial hypertension and dilation and/or failure of the right ventricle. The latter yields a release of troponin and BNP. According to the literature, serum troponin is one of the significant predictors in PE [10,24]. Previous investigations showed that an elevated troponin level was associated with right ventricular dysfunction in PE [24,25]. Furthermore, numerous reports suggested that troponin level was an independent predictor of short-term outcome in patients with PE [10,24,25]. In addition, troponin can predict long-term outcome in PE [10,24,25]. These results were confirmed by a meta-analysis, which found that elevated troponin levels were associated with a five-fold increased risk for all causes of short-term mortality and about a four-fold increased risk for serious adverse events [10]. Previously, only three studies with relatively small numbers of patients analyzed associations between troponin level and pulmonary obstruction in pulmonary embolism patients [26,27,28]. Furthermore, the reported correlation coefficients were weak. Thieme et al. [26], in their study with 63 patients, found a statistically significant correlation between Mastora score and troponin level (*r =* 0.37, *p* = 0.016). Furthermore, in the study of Gül et al. [27] which investigated 28 patients with PE, vessel obstruction index (Qanadli score) correlated slightly with troponin level (*r =* 0.32, *p* = 0.01). Similarly, Jeebun et al. [28] showed a higher statistically significant correlation between clot burden and troponin level in patients (*r =* 0.41, *p* = 0.048). In the present study, no significant correlation between troponin level and Mastora score was observed. Therefore, it may be postulated that thrombotic vessel obstruction cannot be considered a main cause of cardiac damage in PE. 

Additionally, BNP was reported as a significant predictor of clinical outcome in patients with PE [29]. Surprisingly, there were no studies investigating associations between clot burden and BNP. As shown, clot burden tended to correlate with BNP level. However, the correlation coefficient was weak. This finding suggests that vessel obstruction also does not appear to be a major factor of cardiac dilatation in PE. 

According to the literature, there are also other parameters that can predict the severity and prognosis of PE. For instance, serum lactate has been reported as a powerful predictor of short-term PE-related complications [30]. Moreover, patients with high lactate values had a higher mortality rate [8,30]. No previous investigations analyzed associations between lactate and clot burden in PE. As shown in the present study, Mastora score only correlated weakly with serum lactate value, i.e., the relationship between the parameters is not linear. Therefore, it can be postulated that, paradoxically, vessel obstruction does not play a central role in tissue hypoxia. 

Overall, the present study showed that the mechanisms of heart dysfunction and/or failure in PE are more complex than was assumed and cannot be explained by only mechanical vessel obstruction.

The present study is limited to its retrospective design. In order to reduce possible bias, the CTs were evaluated in a blinded manner in relation to the clinical features. However, as mentioned above, this is the first study to investigate direct associations between thrombus burden and the established clinically relevant parameters in PE. Furthermore, this study investigated a large number of patients. Clearly, further prospective studies are needed to confirm our results.

In conclusion, our study showed that thrombotic obstruction of pulmonary vessels did not correlate with clinical severity of PE.

## Figures and Tables

**Table 1:** Analysis of clinical and radiological parameters.

Parameters	Mean ± Standard Deviation	Median	Range
Wells score	6.06 ± 1.79	6	3–10.5
Geneva score	13.61 ± 3.57	7	0–17
D-dimer	13.10 ± 14.84	7.21	0.26–128
PESI score	2 ± 1	2	0–6
Troponin	90.69 ± 141.98	55.18	3–128
BNP	2112.25 ± 2622.85	776.7	0–10,445
Lactate	3.48 ± 3.56	2.3	0.6–23
pH	7.34 ± 0.15	7.39	6.59–7.53
Mastora score	77.49 ± 26.04	82	11–137

PESI: pulmonary embolism severity index; BNP: N-terminal natriuretic peptide.

**Table 2:** Correlations between Mastora score and clinical/serological parameters in patients with pulmonary embolism.

	Wells Score	Geneva Score	D-Dimer	PESI Score	BNP	Troponin	Lactate	pH
Mastora Score	ρ = 0.07	ρ = 0.06	ρ = 0.15	ρ = -0.03	ρ = 0.29	ρ = 0.08	ρ = 0.17	ρ = 0.02
	*p* = 0.27	*p* = 0.40	*p* = 0.09	*p* = 0.39	*p* = 0.063	*p* = 0.29	*p* = 0.01	*p* = 0.77
